# Are Strategies Favoring Pattern Matching a Viable Way to Improve Complexity Estimation Based on Sample Entropy?

**DOI:** 10.3390/e22070724

**Published:** 2020-06-30

**Authors:** Alberto Porta, José Fernando Valencia, Beatrice Cairo, Vlasta Bari, Beatrice De Maria, Francesca Gelpi, Franca Barbic, Raffaello Furlan

**Affiliations:** 1 Department of Biomedical Sciences for Health, University of Milan, 20133 Milan, Italy; beatrice.cairo@unimi.it; 2 Department of Cardiothoracic, Vascular Anesthesia and Intensive Care, IRCCS Policlinico San Donato, San Donato Milanese, 20097 Milan, Italy; vlasta.bari@grupposandonato.it (V.B.); francesca.gelpi@grupposandonato.it (F.G.); 3 Department of Electronic Engineering, Universidad de San Buenaventura, Cali 760033, Colombia; jfvalenc@usbcali.edu.co; 4 IRCCS Istituti Clinici Scientifici Maugeri, 20138 Milan, Italy; beatrice.demaria@icsmaugeri.it; 5 Department of Internal Medicine, IRCCS Humanitas Clinical and Research Center, Humanitas University, 20089 Rozzano, Italy; franca.barbic@humanitas.it (F.B.); raffaello.furlan@hunimed.eu (R.F.)

**Keywords:** conditional entropy, information dynamics, time series analysis, heart rate variability, systolic blood pressure, cardiovascular control, autonomic nervous system, head-up tilt, Parkinson disease

## Abstract

It has been suggested that a viable strategy to improve complexity estimation based on the assessment of pattern similarity is to increase the pattern matching rate without enlarging the series length. We tested this hypothesis over short simulations of nonlinear deterministic and linear stochastic dynamics affected by various noise amounts. Several transformations featuring a different ability to increase the pattern matching rate were tested and compared to the usual strategy adopted in sample entropy (SampEn) computation. The approaches were applied to evaluate the complexity of short-term cardiac and vascular controls from the beat-to-beat variability of heart period (HP) and systolic arterial pressure (SAP) in 12 Parkinson disease patients and 12 age- and gender-matched healthy subjects at supine resting and during head-up tilt. Over simulations, the strategies estimated a larger complexity over nonlinear deterministic signals and a greater regularity over linear stochastic series or deterministic dynamics importantly contaminated by noise. Over short HP and SAP series the techniques did not produce any practical advantage, with an unvaried ability to discriminate groups and experimental conditions compared to the traditional SampEn. Procedures designed to artificially increase the number of matches are of no methodological and practical value when applied to assess complexity indexes.

## 1. Introduction

In time series analysis, complexity markers are frequently computed to estimate the degree of irregularity in dynamics. For example, in cardiovascular control studies [1,2,3,4], the complexity of spontaneous fluctuations of heart period (HP) and systolic arterial pressure (SAP) is routinely assessed under the hypothesis that in pathological conditions the number of interacting control mechanisms responsible for the regulation of cardiovascular variables is significantly decreased and/or weakened leading to a decrease in the overall complexity of the HP and SAP series [5,6,7]. Complexity is usually assessed in the information domain via model-free approaches estimating the conditional entropy (CE) according to methods based on the assessment of the probability of finding similar patterns, usually referred to as matches. Among these techniques, approximate entropy and its refinements, sample entropy (SampEn) and corrected CE [8,9,10,11], play an important role. In short-term cardiovascular control analysis, these tools are widely applied for their ability to deal with short and noisy HP and SAP series (i.e., about 5 min) [11,12,13,14,15,16,17,18,19,20,21,22,23,24]. When addressing short-term autonomic regulation, it has been recommended to keep the length of the series constant and short in such a way as to address the proper range of time scales and avoid confounding factors associated with the activity of slower mechanisms (e.g., humoral regulation) [25,26,27]. Regardless of the application of complexity analysis, the use of short data sequences is generally privileged over long series because it helps the fulfillment of the requirement of stationarity, being the typical prerequisite for the application of tools assessing complexity based on probability estimation of pattern occurrences like CE [28]. One of the major limitations of model-free complexity markers based on the concept of pattern similarity lies in the reliability of the pattern matching rate when calculated over such short series. Recently, some workarounds have been proposed to increase similarity among patterns and limit the dispersion of the patterns about the reference [29,30,31]. This issue becomes dramatic, especially when the pattern length is large, because the degree of dissimilarity among patterns increases more and more with their duration or, equivalently, patterns become more and more sparse while enlarging the dimension of the embedding space in which the dynamics are reconstructed [32]. Therefore, increasing the number of matches without augmenting the series length has been proposed as a viable possibility to improve the robustness of the approximation of probability with sample frequency and to limit random fluctuations of the complexity index [29]. These workarounds are mainly based on transformations of the current pattern such a way that its similarity to the reference pattern is found not only when the two original patterns are similar but also when the two original patterns are dissimilar and become similar after transformation.

The aim of this study was to test these workarounds over short simulated series simulating partially predictable dynamics corrupted by increasing amounts of noise [24] and in the practical context of assessing cardiovascular control complexity in healthy (H) subjects and Parkinson disease (PD) patients [33,34]. This contribution is organized as follows: in the Methods Section we describe the strategies exploited to increase the number of matched pairs and how these strategies were applied in SampEn computation; in the Simulated Data and Nonlinearity Test Section we describe the simulated partially predictable signals, how the original realizations were corrupted by noise and how the presence of nonlinear dynamics was checked; in the Experimental Protocol and Data Analysis Section we describe our experimental protocol and series extraction; in the Results, Discussion and Conclusions we summarize the results, interpret the findings and draw the conclusions of the study, respectively.

## 2. Methods

### 2.1. Pattern Definition, Transformations and Matching

We assign the series *x* = {$x_{n}$, *n* = 1, …, *N*}, where *n* is the progressive counter and *N* is the series length; *x* is first normalized by subtracting the mean and by dividing each resulting value by the standard deviation, thus obtaining a normalized series fluctuating about 0 with unit variance. Given the current value $x_{n}$, we consider the pattern $x_{n}^{-}=[\begin{array}{ccc} x_{n-1} & \cdots & x_{n-m+1} \end{array}]$ formed by (*m* − 1) consecutive past values of $x_{n}$ and the pattern $x_{n}=[\begin{array}{cccc} x_{n} & x_{n-1} & \cdots & x_{n-m+1} \end{array}]$ obtained by concatenating $x_{n}$ to $x_{n}^{-}$. The patterns $x_{n}^{-}$ and $x_{n}$ are points in (*m* − 1)-dimensional and *m*-dimensional embedding spaces built with the technique of time-delayed coordinates [35].

We will consider the following transformations [29,30,31] applied to patterns: (i) centering *C*(·) subtracts from each component of the pattern the mean assessed over it, namely *C*($x_{n}$) = $[\begin{array}{cccc} \Delta x_{n} & \Delta x_{n-1} & \cdots & \Delta x_{n-m+1}] \end{array}$, where Δ represents the deviation of each coordinate of the pattern from its mean; (ii) inversion *I*(·) switches the sign of all pattern components by multiplying each coordinate by −1, namely *I*($x_{n}$) = $[\begin{array}{cccc} -x_{n} & -x_{n-1} & \cdots & -x_{n-m+1}] \end{array}$; (iii) time reversal *R*(·) reverts the flow of time within a given pattern in such a way that the most delayed sample becomes the first component and the most recent sample becomes the last component, namely *R*($x_{n}$) = $[\begin{array}{cccc} x_{n-m+1} & \cdots & x_{n-1} & x_{n}] \end{array}$; (iv) inversion after time reversal *IR*(·) = *I*(*R*(·)) inverts the reverted pattern, namely *I*(*R*($x_{n}$)) = *I*($[\begin{array}{cccc} x_{n-m+1} & \cdots & x_{n-1} & x_{n}] \end{array}$) = $[\begin{array}{cccc} {-x}_{n-m+1} & \cdots & -x_{n-1} & -x_{n}] \end{array}$. It is worth noting that the time reversal applied after inversion, namely *RI*(·) = *R*(*I*(·)), leading to reversion of the time flow of the inverted pattern, provides the same result as *IR*(·). Let us consider, as an example, the sine function $x_{n}$ = sine(2π·*n*·*f*/*f_s_*), where *f*/*f_s_* represents the ratio of the frequency *f* of the sine to the sampling frequency *f_s_* with *f*/*f_s_* = 1/50. Assigned the pattern $x_{7}$ = $\left[\begin{array}{cc} x_{7} & x_{6} \end{array}]=[\begin{array}{ccc} 0.77 & & 0.68 \end{array}\right]$ on the ascending phase of the first positive half cycle of the sine curve, *I*($x_{7}$) = $\left[\begin{array}{ccc} -0.77 & & -0.68 \end{array}\right]$ = $x_{32}$ lies on the descending part of the first negative half cycle of the sine curve, *R*($x_{7}$) = $\left[\begin{array}{ccc} 0.68 & & 0.77 \end{array}\right]$ = $x_{19}$ lies on the descending part of the first positive half cycle of the sine curve, and *IR*($x_{7}$) = $\left[\begin{array}{ccc} -0.68 & & -0.77 \end{array}\right]$ = $x_{44}$ lies on the ascending part of the first negative half cycle of the sine curve.

Two patterns are matched if the distance between them is smaller than *r*, namely ‖$x_{n}$ − $x_{m}$‖ < *r*, and *r* is usually referred to tolerance and ‖·‖ is an assigned norm (e.g., Euclidean norm) [8,10].

### 2.2. SampEn Computation

SampEn assesses the complexity of *x* via the computation of the amount of information associated with $x_{n}$, which cannot be derived from $x_{n}^{-}$ (i.e., the CE). SampEn is computed [10] as

(1) $$\mathrm{SampEn}(m,r,N)=-\log\frac{\langle p(x_{n})\rangle }{\langle p(x_{n}^{-})\rangle },$$

where $p(x_{n})$ and $p(x_{n}^{-})$ represent, respectively, the probability of finding a pattern in the neighborhood of $x_{n}$ and $x_{n}^{-}$ of size *r*, computed by dividing the number of matches of $x_{n}$ and $x_{n}^{-}$ by the correspondent total number of patterns, and the operator <·> performs the mean over the time index *n*. After assigning *r* and *N*, increasing the number of matches could improve the robustness of the estimate of $p(x_{n})$ and $p(x_{n}^{-})$ because sample frequencies should become less sensible to small variations in the number of matches owing to the smallness of *N* and to the strict boundary condition in the definition of the neighborhood of $x_{n}$ and $x_{n}^{-}$.

### 2.3. Strategies for Increasing the Number of Matches

Assigned the reference pattern $x_{n}$, the pattern $x_{m}$ is matched to $x_{n}$ by one of the following strategies [29]: (i) $x_{m}$ is closer than *r* to $x_{n}$ according to the traditional definition of SampEn, and this strategy is labeled S; (ii) $x_{m}$, or *I*($x_{m}$), is closer than *r* to $x_{n}$ and this strategy is labeled SI; (iii) $x_{m}$, or *R*($x_{m}$), is closer than *r* to $x_{n}$ and this strategy is labeled SR; (iv) $x_{m}$, or *I*($x_{m}$), or *R*($x_{m}$), is closer than *r* to $x_{n}$ and this strategy is labeled SIR; (v) $x_{m}$, or *I*($x_{m}$), or *R*($x_{m}$), or *IR*($x_{m}$), is closer than *r* to $x_{n}$ and this strategy is labeled SIR2. To summarize, $x_{n}$ and $x_{m}$ are matched for the following:

(2) $$S:\;\|x_{n}-x_{m}\|<r,\;\mathrm{SI}:\;\|x_{n}-x_{m}\|<r\;\mathrm{or}\;\|x_{n}-I(x_{m})\|<r,\;\mathrm{SR}:\;\|x_{n}-x_{m}\|<r\;\mathrm{or}\;\|x_{n}-R(x_{m})\|<r,\;\mathrm{SIR}:\;\|x_{n}-x_{m}\|<r\;\mathrm{or}\;\|x_{n}-I(x_{m})\|<r\;\mathrm{or}\;\|x_{n}-R(x_{m})\|<r,\;\mathrm{SIR}2:\;\|x_{n}-x_{m}\|<r\;\mathrm{or}\;\|x_{n}-I(x_{m})\|<r\;\mathrm{or}\;\|x_{n}-R(x_{m})\|<r\;\mathrm{or}\;\|x_{n}-IR(x_{m})\|<r.$$

The use of SI and SR strategies leads to an increase in the number of matches compared to S. The use of SIR and SIR2 strategies leads to an increase in the number of matches compared to SI and SR, with the number of matches of SIR2 larger than that of SIR. The abovementioned strategies can be applied over centered patterns [29,30,31] *C*($x_{n}$) and *C*($x_{m}$), thus leading to centered S (CS), centered CI (CSI), centered SR (CSR), centered SIR (CSIR) and centered SIR2 (CSIR2) strategies. CS, CSI, CSR, CSIR and CSIR2 increase the number of matches compared to S, SI, SR, SIR and SIR2, respectively, because only the shapes of the patterns are considered regardless of their mean values [30,31]. To summarize the centered strategies, $x_{n}$ and $x_{m}$ are matched for the following:

(3) $$\mathrm{CS}:\;\|C(x_{n})-C(x_{m})\|<r,\;\mathrm{CSI}:\;\|C(x_{n})-C(x_{m}))\|<r\;\mathrm{or}\;\|C(x_{n})-I(C(x_{m}))\|<r,\;\mathrm{CSR}:\;\|C(x_{n})-C(x_{m})\|<r\;\mathrm{or}\;\|C(x_{n})-R(C(x_{m}))\|<r,\;\mathrm{CSIR}:\;\|C(x_{n})-C(x_{m})\|<r\;\mathrm{or}\;\|C(x_{n})-I(C(x_{m}))\|<r\;\mathrm{or}\;\|C(x_{n})-R(C(x_{m}))\|<r,\;\mathrm{CSIR}2:\;\|C(x_{n})-C(x_{m}))\|<r\;\mathrm{or}\;\|C(x_{n})-I(C(x_{m}))\|<r\;\mathrm{or}\;\|C(x_{n})-R(C(x_{m}))\|<r\;\mathrm{or}\;\|C(x_{n})-IR(C(x_{m}))\|<r.$$

**Figure 1:**
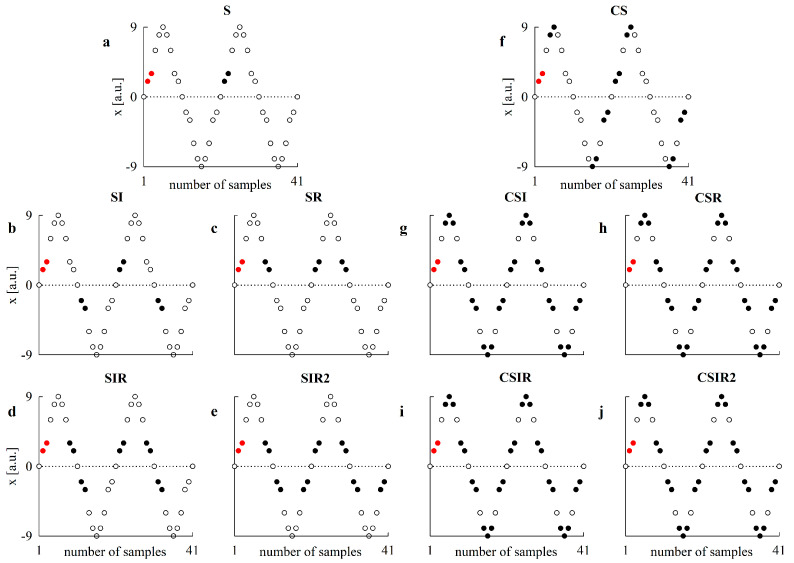
Examples of matches with tolerance *r* = 0 in a periodic repetition of the pattern [0, 2, 3, 6, 8, 9, 8, 6, 3, 2, 0, −2, −3, −6, −8, −9, −8, −6, −3, −2] are given as a function of the pattern matching strategy. The reference pattern is denoted with red solid circles and its matches with tolerance *r* = 0 are indicated with black solid circles. The reference pattern and its matches are shown with the S strategy in (**a**), SI strategy in (**b**), SR strategy in (**c**) SIR strategy in (**d**), SIR2 strategy in (**e**), CS strategy in (**f**), CSI strategy in (**g**), CSR strategy in (**h**), CSIR strategy in (**i**) and CSIR2 strategy in (**j**).

Figure 1 shows examples of matches with tolerance *r* = 0 according to the different pattern matching strategies in a synthetic periodic signal. The signal is a periodic repetition of the pattern [0, 2, 3, 6, 8, 9, 8, 6, 3, 2, 0, −2, −3, −6, −8, −9, −8, −6, −3, −2]. The reference pattern is shown as red solid circles and any matched pattern with tolerance *r* = 0 is shown as a black solid circle as a function of the pattern matching strategy. Figure 1a–e show the result of the application of S, SI, SR, SIR and SIR2 strategies and Figure 1f–j show the result of the application of CS, CSI, CSR, CSIR and CSIR2 strategies. Comparison among S, SI, SR, SIR and SIR2 strategies (Figure 1a–e) suggests that the number of matches rises with the complexity of the matching pattern strategy. Moreover, matched patterns might belong to different phases of the periodic signal, and this feature is more evident with more complex pattern matching strategies. Comparison among CS, CSI, CSR, CSIR and CSIR2 strategies (Figure 1f–j) indicates that the centered pattern strategies notably increase the number of matches and an important rise is already evident with the simplest centered pattern matching strategies and obviously preserved by the most complex ones.

## 3. Simulated Data and Nonlinearity Test

### 3.1. Simulations

We considered two types of dynamics [24]: (i) a realization of deterministic chaos generated with the logistic map, namely $x_{n}=k\cdot x_{n-1}\cdot (1-x_{n-1})$ with *k* = 3.7 (type-I simulation); (ii) a realization of a stochastic linear process with a dominant spectral peak generated via a second-order autoregressive model featuring two complex and conjugate poles with modulus *ρ* = 0.92 and phases *φ* = ±π/5 and driven by Gaussian white noise with zero mean and unit variance (type-II simulation). The dynamic generated by the logistic map in the chaotic regime was chosen because of its nonlinear properties [24] and different behavior under time reversal [36], both properties present in heart rate variability [37,38]. The dynamic generated by the autoregressive process was chosen because of its fully linear properties and invariable behavior under time reversal [39], these being features typical of stochastic components of heart rate variability [38]. The ability of SampEn in connection with the various strategies adopted for increasing the pattern matching rate in dealing with broadband noise was assessed by adding independent, identically distributed white noise to the initially simulated signals (uncorrupted series). The noise realizations had zero mean and standard deviation set as a percentage of the standard deviation of the uncorrupted series. The standard deviation of noise increased starting from 1% in steps of 2% (i.e., 1%, 3%, 5%, ···) until 59% was reached. We generated 50 realizations of contaminated series for each level of superimposed noise by randomly changing the seed of the white noise. The course between the 2.5th and 97.5th percentiles of SampEn as a function the percentage of noise computed over the set of simulated signals according to the different pattern matching strategies was superimposed on the one calculated using the S strategy. The effect of the pattern matching strategies relative to the S strategy on SampEn was directly assessed over the simulated series corrupted by the minimal amount of noise (i.e., 1%).

### 3.2. Surrogate Series and Detection of Nonlinear Dynamics

We tested the null hypothesis that type-I simulations corrupted by noise are realizations of a linear process with a Gaussian distribution, possibly distorted via a nonlinear static invertible transformation. According to this null hypothesis, we built surrogate series with the same second-order statistical properties (i.e., with preserved a power spectrum) and the same distribution (i.e., with a preserved histogram) as the original ones but with random phases via iterated amplitude-adjusted Fourier transform procedure [40,41]. Fourier phases were drawn from a uniform distribution bounded between 0 and 2π. The number of iterations to achieve the best approximation of the original power spectrum with the exact distribution of values of the original series was fixed to 100 [41]. We constructed one surrogate for each original realization. If nonlinear features were present in the original series, the SampEn computed over the original series would be smaller than that computed over linear surrogates. Therefore, the distribution of SampEn was computed over the original and surrogate series and the two distributions were compared. The null hypothesis was rejected and the alternative hypothesis (i.e., data were generated by a nonlinear dynamical system) was accepted when the 97.5th percentile of SampEn computed over the original series was found to be below the 2.5th percentile of SampEn computed over the surrogate data (i.e., the original series were significantly less complex than surrogates) [24]. The different strategies adopted to increase the number of matches were exploited in the computation of SampEn, and the results of the nonlinearity test were discussed as a function of the pattern matching strategy.

## 4. Experimental Protocol and Data Analysis

### 4.1. Experimental Protocol

The protocol was originally designed to typify cardiovascular control and its complexity in PD patients through HP and SAP variability analyses [33,34]. Briefly, we studied 12 patients with PD without orthostatic hypotension or symptoms of orthostatic intolerance (age range: 55–79 years; median: 65 years; 8 men) and 12 H subjects matched by age and gender with those in the PD group (age range: 58–72 years; median: 67 years; 7 men). PD patients (Hoehn and Yahr scale: stages 2–4) were at the best of their habitual pharmacological treatment. Electrocardiograms (ECG) from lead II and noninvasive arterial pressure (Finapress 2300, Ohmeda, Englewood, CO) were recorded. Sample frequency was 300 Hz. Signals were recorded for 10 min at rest in supine condition (REST) and during head-up tilt with table inclination set at 75° (HUT). All subjects gave their written informed consent. The study adhered to the principles of the Declaration of Helsinki for medical research involving human subjects. The protocol was approved by the ethical review board of the Bolognini Hospital of Seriate, Bergamo, Italy (project identification code: 493, approval date: 15-6-2011). The experimental protocol and instrument types are standard in the field of cardiovascular control assessment based on spontaneous fluctuations of physiological variables [2,4].

### 4.2. Extraction of the Beat-to-Beat Variability and Preprocessing Techniques

After detecting the QRS complex using a traditional method based on a threshold on the first derivative of the ECG and locating the R-wave peak with minimum jitters using parabolic interpolation, the temporal distance between two consecutive QRS apexes was computed and utilized as an approximation of the *n*th HP (HP*_n_*). The maximum arterial pressure within HP*_n_* was taken as the *n*th SAP (SAP*_n_*). Fiducial points were carefully checked to avoid erroneous detections or missed beats. If isolated ectopic beats affected HP and SAP values, these measures were linearly interpolated using the closest values unaffected by ectopic beats. Sequences of 256 consecutive HP and SAP values were randomly selected within REST and HUT sessions. The procedures for the extraction of physiological variables, strategy for correction of artifacts and duration of the frame are standard in short-term heart rate variability analysis [3,25]. Time domain indexes have already been reported [34]: briefly, (i) the HP mean decreased during HUT in both H and PD individuals, but no between-group difference was observed regardless of the experimental condition; (ii) HP variance and SAP mean were similar regardless of the group and experimental condition; (iii) SAP variance increased during HUT exclusively in H subjects and was smaller in PD patients compared to H individuals during HUT.

### 4.3. Assessing Complexity Using the Different Matching Strategies

SampEn was calculated according to the standard settings [10,23,31], namely, *m* = 2, *r* = 0.2 × the standard deviation of the series, *N* = 256, and Euclidean norm, to calculate distances among the patterns. SampEn was computed over simulated and real data after linear detrending. SampEn was calculated according to the different strategies designed to increase the number of matches, namely S, SI, SR, SIR and SIR2 and CS, CSI, CSR, CSIR and CSIR2. The difference between the 97.5th and the 2.5th percentiles of SampEn was taken as a measure of the SampEn dispersion about the median. This index was computed for all the strategies, and it was divided by that calculated via the S strategy, thus quantifying the variation of the SampEn variance induced by the adopted pattern matching approach compared to the S strategy. This ratio was labeled the variance reduction ratio (VRR). If the VRR was significantly below 1, the considered strategy reduced the variance of SampEn. The assessment of VRR was carried out over simulated signals and real series.

### 4.4. Statistical Analysis

One-way repeated measures analysis of variance, or Friedman repeated measures analysis of variance on ranks when appropriate, was applied (Tukey’s test for multiple comparisons) to check whether the different strategies for the assessment of matches affected SampEn computed over type-I and type-II simulations corrupted by a minimal amount of noise (i.e., 1% of noise). Two-way repeated measures analysis of variance (one-factor repetition, Holm–Sidak test for multiple comparisons) was performed to assess the significance of SampEn changes induced by the orthostatic challenge within the same population (H or PD group) and by the pathology within the same experimental condition (REST or HUT). The analysis was repeated with different pattern matching strategies to check for discrepancies among conclusions. Statistical analysis was carried out using a commercial statistical program (Sigmaplot, v.14.0, Systat Software, Inc., Chicago, IL, USA). A type-I error probability *p* < 0.05 was always considered significant.

## 5. Results

### 5.1. Simulated Type-I and Type-II Series: Effect of Pattern Matching Strategies on SampEn

**Figure 2:**
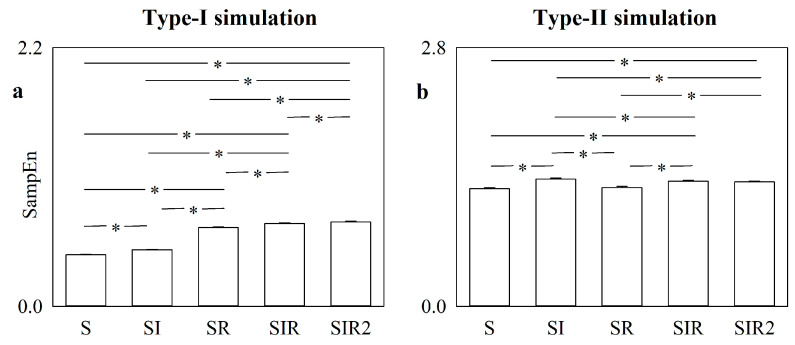
The error bar graphs show the SampEn computed over type-I (**a**) and type-II (**b**) simulations affected by independent identically distributed white noise with standard deviation equal to 1% of the standard deviation of the uncorrupted dynamic. Values are given as a function of the strategy exploited to increase the number of matches, namely S, SI, SR, SIR, or SIR2. Data are reported as mean plus standard deviation. The symbol * indicates *p* < 0.05.

The error bar graphs in Figure 2 show SampEn computed over type-I (Figure 2a) and type-II (Figure 2b) simulations contaminated with independent identically distributed white noise with standard deviation equal to 1% of the standard deviation of the uncorrupted chaotic and autoregressive series as a function of the strategy exploited to find matched patterns (i.e., S, SI, SR, SIR and SIR2).

In the case of type-I simulation (Figure 2a), the smallest value of SampEn was detected when the marker was computed according to the original strategy of detecting matched patterns, while the application of any alternative strategy limited the ability of past values to predict future behaviors and increased SampEn. A progressive increase in SampEn was observed passing from S to SI, from SI to SR, from SR to SIR and, finally, from SIR to SIR2. The most relevant rise was visible when pattern matching was tested after time reversal transformation via SR, SIR and SIR2 approaches compared to S and SI, and this result is linked to the irreversible nature of the logistic map dynamics in the chaotic regime. The results of SampEn obtained from type-II simulation (Figure 2b) also showed that the smallest SampEn was calculated with the S strategy. However, SampEn values were more homogeneous across pattern matching strategies. Remarkably, no significant difference was detected between SampEn computed using S and SR strategies and between SampEn computed using SIR and SIR2 approaches as a result of the reversible nature of the linear dynamics generated by an autoregressive process.

**Figure 3:**
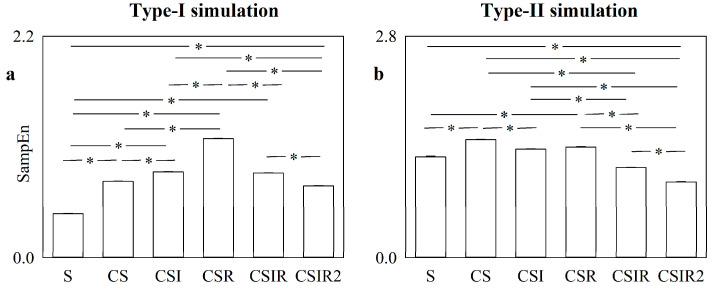
The error bar graphs show the SampEn computed over type-I (**a**) and type-II (**b**) simulations affected by independent identically distributed white noise with standard deviation equal to 1% of the standard deviation of the uncorrupted dynamic. Values are given as a function of the strategy exploited to increase the number of matches, namely S, CS, CSI, CSR, CSIR, or CSIR2. Data are reported as mean plus standard deviation. The symbol * indicates *p* < 0.05.

Figure 3 has the same structure as Figure 2, and SampEn was computed over the same simulations, but it shows SampEn as a function of the matching pattern strategies based on the transformations operated over centered patterns (i.e., CS, CSI, CSR, CSIR and CSIR2).

In the case of type-I simulation (Figure 3a), the smallest value of SampEn was again found using the S strategy. The application of the centering transformation via the CS strategy increased SampEn and, similarly to Figure 2a, the rise in SampEn was especially evident using the CSR strategy. Unlike in Figure 2a, further increasing the number of matches via the CSIR and CSIR2 strategies produced a decrease in SampEn compared to CSR. Indeed, the number of matches found at embedding dimension *m* − 1 by CSIR and CSIR2 was so high that the increment at embedding dimension *m* was negligible compared to with the CSR strategy. Results obtained from type-II simulation (Figure 3b) outlined that SampEn computed via the CS strategy is higher than that based on the S one and a similar increase was observed when the CSR strategy was applied. However, unlike with type-I simulation, variations compared to S were less remarkable as an effect of the stochastic linear nature of the autoregressive process compared to the nonlinear deterministic nature of the logistic map (e.g., no difference between CS and CSR was detected). Similarly to type-I simulation, CSIR and CSIR2 strategies led to SampEn significantly smaller than that compared via the CSR strategy.

### 5.2. Simulated Type-I and Type-II Series: The Effect of Pattern Matching Strategies on SampEn Depends on Noise Level

**Figure 4:**
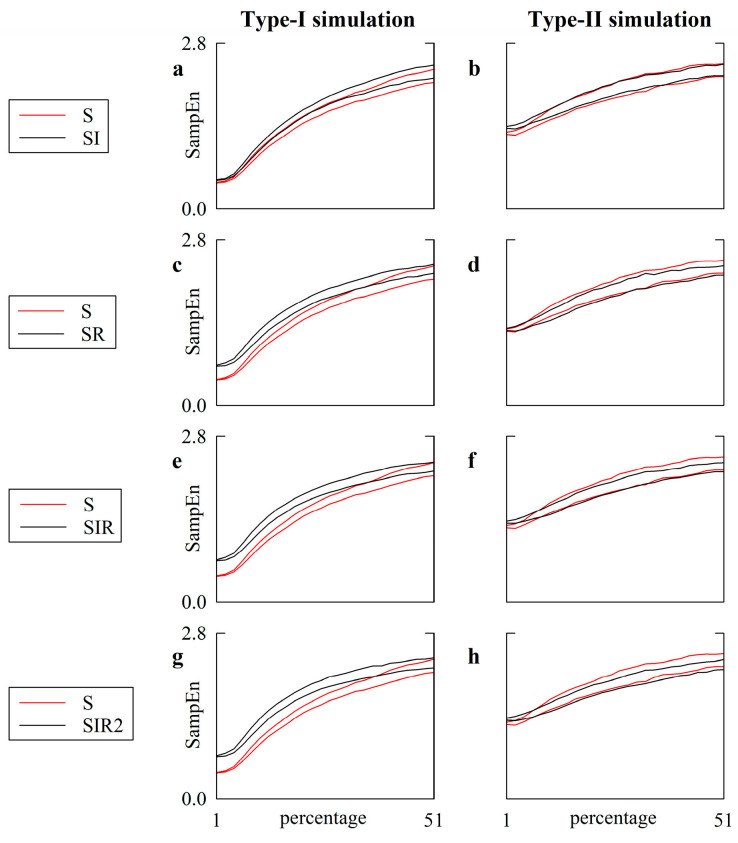
The line plots show the SampEn computed over type-I (**a**,**c**,**e**,**g**) and type-II (**b**,**d**,**f**,**h**) simulations as a function of the amplitude of independent identically distributed white noise superimposed on the uncorrupted dynamics. The amplitude of the white noise is monitored as a percentage of the standard deviation of the uncorrupted series. The solid red lines represent the SampEn computed according to the strategy of pattern matching traditionally exploited in SampEn computation (i.e., S), while the solid black lines are relevant to the various definitions of non-centered strategies adopted in this study, namely SI (**a**,**b**), SR (**c**,**d**), SIR (**e**,**f**) and SIR2 (**g**,**h**). The two lines are relevant to the 2.5th and 97.5th percentiles computed over the set of simulations.

Figure 4 provides the comparison between SampEn derived according to the SI (Figure 4a,b), SR (Figure 4c,d), SIR (Figure 4e,f) and SIR2 (Figure 4g,h) strategies (solid black lines) and the S strategy (solid red lines) as a function of the amplitude of the white noise contaminating the chaotic and autoregressive dynamics. The level of noise was monitored as a percentage of the standard deviation of the uncorrupted chaotic and autoregressive series. Results are relevant to type-I (Figure 4a,c,e,g) and type-II (Figure 4b,d,f,h) simulations. The two (black or red) lines are relevant to the 2.5th and 97.5th percentiles of SampEn computed over the set of 50 simulations.

In the case of the type-I simulation, the SampEn calculated via the S strategy was below that computed through SI, SR, SIR and SIR2, and this result was robust until the percentage of noise was smaller than, respectively, 7, 33, 37 and 39. Indeed, in correspondence with these percentages, the 97.5th percentile of SampEn computed using S became larger than the 2.5th percentile of SampEn calculated using the SI, SR, SIR and SIR2 strategies respectively. In the case of type-II simulation, the 97.5th percentile of SampEn computed using S surpassed the 2.5th percentile of SampEn calculated using SI, SR, SIR and SIR2 at percentages of 7, 1, 5 and 3, respectively. In both type-I and type-II simulations, the SampEn computed using S did not significantly rise above the SampEn computed via the SI, SR, SIR and SIR2 strategies, given that the 2.5th percentile of SampEn computed according to S never surpassed the 97.5th percentile of SampEn calculated via the SI, SR, SIR and SIR2 strategies. These results suggest that differences among SampEn imposed by the exploitation of the different pattern matching strategies became irrelevant compared to the S strategy while increasing the amount of noise, and the rate of this process was faster in a stochastic linear process than in a nonlinear deterministic one. When the type-I simulation was considered, the VRR of SI, SR, SIR and SIR2 averaged over all levels of noise was close to 1 (i.e., 0.96, 0.94, 0.93 and 0.98 respectively). The departure of the mean VRR from 1 was more evident in the case of the type-II simulation, with the mean VRR equal to 0.81, 0.85, 0.76, and 0.77, respectively.

**Figure 5:**
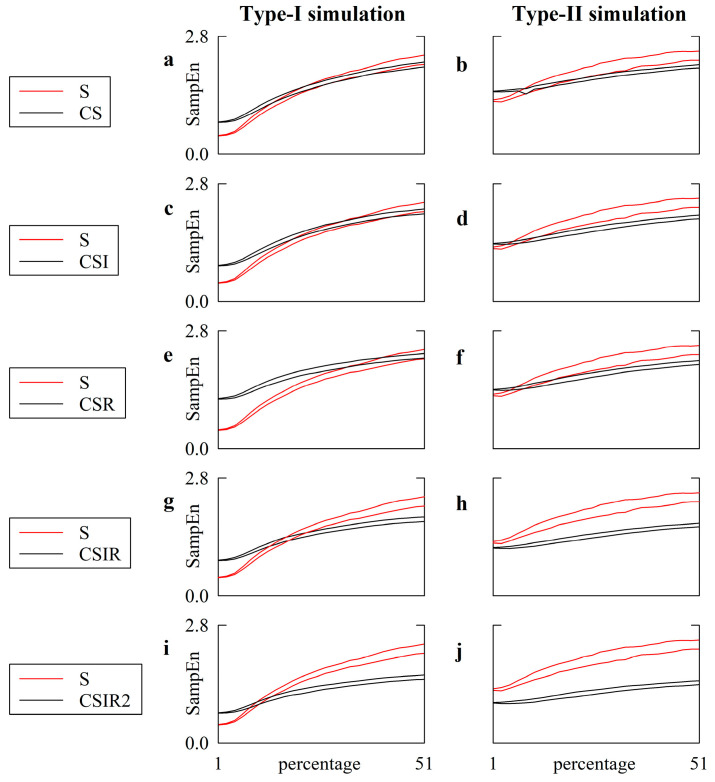
The line plots show SampEn computed over type-I (**a**,**c**,**e**,**g**,**i**) and type-II (**b**,**d**,**f**,**h**,**j**) simulations as a function of the amplitude of independent, identically distributed white noise superimposed on the uncorrupted dynamics. The amplitude of the white noise is monitored as a percentage of the standard deviation of the uncorrupted series. The solid red lines represent SampEn computed according to the strategy of pattern matching traditionally exploited in SampEn computation (i.e., S), while the solid black lines are relevant to the various definition of centered strategies adopted in this study, namely, CS (**a**,**b**), CSI (**c**,**d**), CSR (**e**,**f**), CSIR (**g**,**h**) and CSIR2 (**i**,**j**). The two lines are relevant to the 2.5th and 97.5th percentiles computed over the set of simulations.

Figure 5 has the same structure as Figure 4, but SampEn is computed according to transformations operating over centered patterns, namely CS (Figure 5a,b), CSI (Figure 5c,d), CSR (Figure 5e,f), CSIR (Figure 5g,h) and CSIR2 (Figure 5i,j) strategies. SampEn calculated according to centered strategies (solid black lines) was compared to that computed with the S strategy (solid red lines).

In the case of the type-I simulation (Figure 5a,c,e,g,i) the 97.5th percentile of the SampEn computed using S surpassed the 2.5th percentile of the SampEn calculated using CS, CSI, CSR, CSIR and CSIR2, at percentages of 15, 19, 33, 15 and 9. When further increasing the amount of noise, the SampEn computed through the CS, CSI, and CSR strategies became indistinguishable from the SampEn calculated via S. In the case of the CSIR and CSIR2 strategies, the 2.5th percentile of the SampEn computed using S surpassed the 97.5th percentile of the SampEn assessed via the CSIR and CSIR2 approaches at percentages of 23 and 15. These results suggest that not only could the application of pattern matching strategies operating over centered patterns lead to a SampEn indistinguishable from those computed via the S strategy, but also to different conclusions at low and high levels of noise (i.e., at low levels of noise, the SampEn computed via the CSIR and CSIR2 approaches was significantly larger than that computed using the S approach, while it became smaller at large noise amplitudes). This effect led to an overall reduction in the dynamical range of SampEn, when the deterministic nonlinear dynamics became progressively linear and stochastic through the corruption of noise.

In the case of the type-II simulation (Figure 5b,d,f,h,j) we observed that: (i) the 97.5th percentile of the SampEn computed via the S approach was only below the 2.5th percentile of the SampEn computed using CS, CSI, and CSR at small noise amplitudes, with percentages below, respectively, 9, 5 and 7; (ii) the SampEn computed via using CS, CSI, and CSR became similar to that computed via the S technique at intermediate noise amplitudes; (iii) the 2.5th percentile of the SampEn computed via the S strategy was above the 97.5th percentile of the SampEn computed using CS, CSI, and CSR at high levels of noise, with percentages above, respectively, 21, 11 and 15. This observation suggests, again, that the effect of a strategy improving the number of matches over centered patterns compared to the S strategy depends on the level of superimposed noise, even over a series, like the linear stochastic series, that does not vary its nature due to noise contamination. Moreover, the SampEn calculated via the S strategy was evidently above those computed through the CSIR and CSIR2 approaches, regardless of the amplitude of the noise, given that the 2.5th percentile of the SampEn computed using the S strategy was always above the 97.5th percentile of the SampEn calculated via the CSIR and CSIR2 techniques, thus suggesting that CSIR and CSIR2 could indicate an erroneously greater regularity linked to an artificial increase in matches. When the type-I simulation was considered, the VRRs of CS, CSI, CSR, CSIR and CSIR2 averaged over all levels of noise were significantly different from 1 (i.e., 0.63, 0.62, 0.67, 0.58 and 0.59, respectively) and even further away from 1 in the case of the type-II simulation (i.e., 0.36, 0.41, 0.44, 0.39 and 0.41, respectively).

### 5.3. Simulated Type-I Series: Effect of Pattern Matching Strategies on the Detection of Nonlinear Dynamics

**Figure 6:**
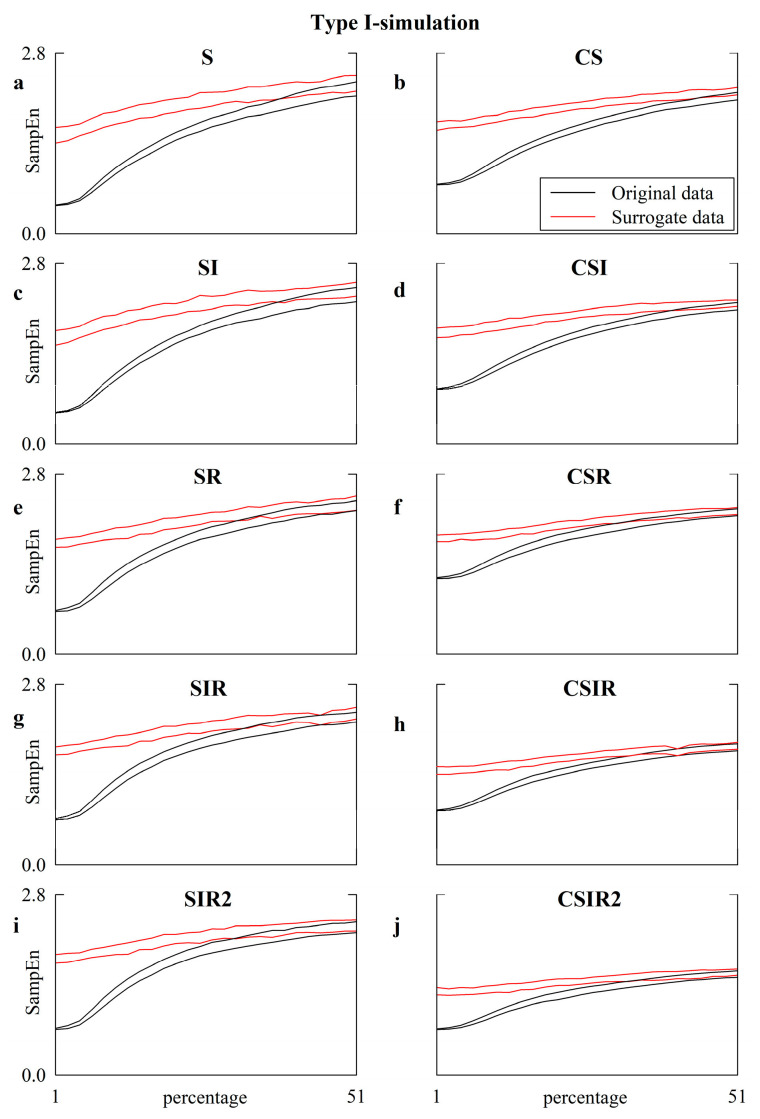
The line plots show SampEn computed over the original (solid black lines) and surrogate (solid red lines) type-I simulations as a function of the amplitude of independent identically distributed white noise superimposed on the dynamic of the uncorrupted series. The amplitude of the white noise is monitored as a percentage of the standard deviation of the uncorrupted series. SampEn is computed according to the strategy of pattern matching adopted in this study, namely S (**a**), CS (**b**), SI (**c**) CSI (**d**), SR (**e**), CSR (**f**), SIR (**g**), CSIR (**h**), SIR2 (**i**), and CSIR2 (**j**). The two lines are relevant to the 2.5th and 97.5th percentiles computed over the set of simulations.

Figure 6 provides a comparison between the SampEn results computed over the original type-I simulations (solid black lines) and their surrogates (solid red lines) according to the S (Figure 6a), CS (Figure 6b), SI (Figure 6c), CSI (Figure 6d), SR (Figure 6e), CSR (Figure 6f), SIR (Figure 6g), CSIR (Figure 6h), SIR2 (Figure 6i) and CSIR2 (Figure 6j) strategies.

The comparison is given as a function of the amplitude of the white noise contaminating the chaotic and autoregressive dynamics expressed as a percentage of the standard deviation of the uncorrupted series. The two (black or red) lines are relevant to the 2.5th and 97.5th percentiles of SampEn computed over the set of the 50 original or surrogate simulations. Nonlinear dynamics were detected until the 97.5th percentile of SampEn assessed over the original simulations surpassed the 2.5th percentile of SampEn computed over the surrogates. This situation was detected at percentages equal to 39, 39, 33, 33 and 31 in the case of S, SI, SR, SIR, and SIR2 approaches, respectively, and at percentages equal to 45, 41, 31, 35 and 33 in the cases of CS, CSI, CSR, CSIR and CSIR2 approaches, respectively, thus remarking that the ability of detecting nonlinear dynamics was negligibly altered by the pattern matching strategies compared to the S strategy. More specifically, the use of the traditional matching strategy over centered patterns (i.e., CS) might even slightly improve the detection performance; namely, nonlinear dynamics were detected with larger levels of noise compared to with S (i.e., 45 vs. 39), while the application of strategies inducing a more notable increase in the number of matches, such as, e.g., SIR, SIR2, CSIR and CSIR2, slightly reduced the ability to detect nonlinear dynamics relative to the S approach (i.e., 33, 31, 35, and 33, respectively, vs. 39).

### 5.4. Real HP and SAP Series: Impact of Pattern Matching Strategies on SampEn

**Figure 7:**
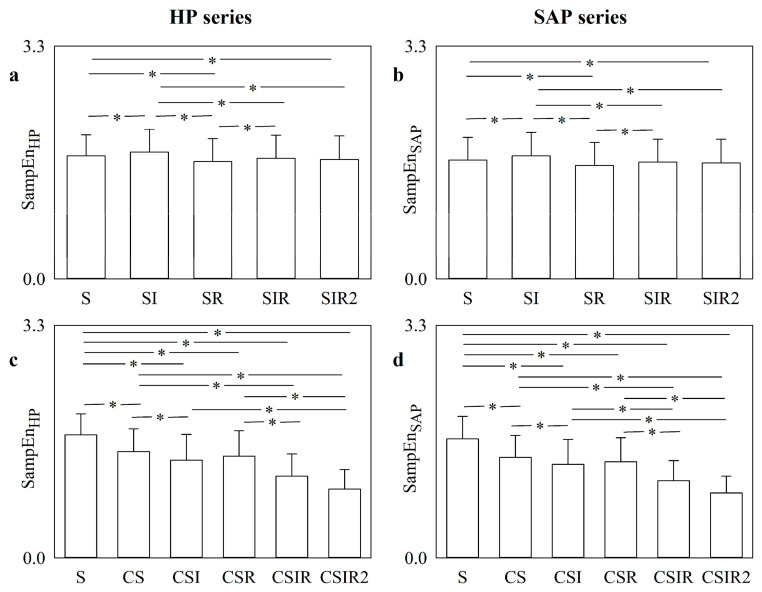
The error bar graphs show SampEn computed over HP (**a**,**c**) and SAP (**b**,**d**) series as a function of the strategy exploited to increase the number of matches, namely S, SI, SR, SIR, and SIR2 in (**a**,**b**) and S, CS, CSI, CSR, CSIR and CSIR2 in (**c**,**d**). Data are reported as mean plus standard deviation. Data are pooled together regardless of the experimental conditions. The symbol * indicates *p* < 0.05.

The error bar graphs in Figure 7 show the SampEn computed over HP (Figure 7a,c) and SAP (Figure 7b,d) series as a function of the pattern matching strategy, namely S, SI, SR, SIR, and SIR2 (Figure 7a,b) and S, CS, CSI, CSR, CSIR and CSIR2 (Figure 7c,d). Data were pooled together regardless of the experimental conditions. Over both HP and SAP series, SampEn varied remarkably as a function of the S, SI, SR, SIR, and SIR2 strategies, with approaches like SI leading to values significantly larger than those computed via the S strategy, and strategies like SR and SIR2 producing values lower than those calculated through the S strategy. Over both HP and SAP series, the applications of the CS, CSI, CSR, CSIR and CSIR2 strategies produced smaller SampEn compared to the S approach, with the smallest value computed by the CSIR and CSIR2 strategies.

**Figure 8:**
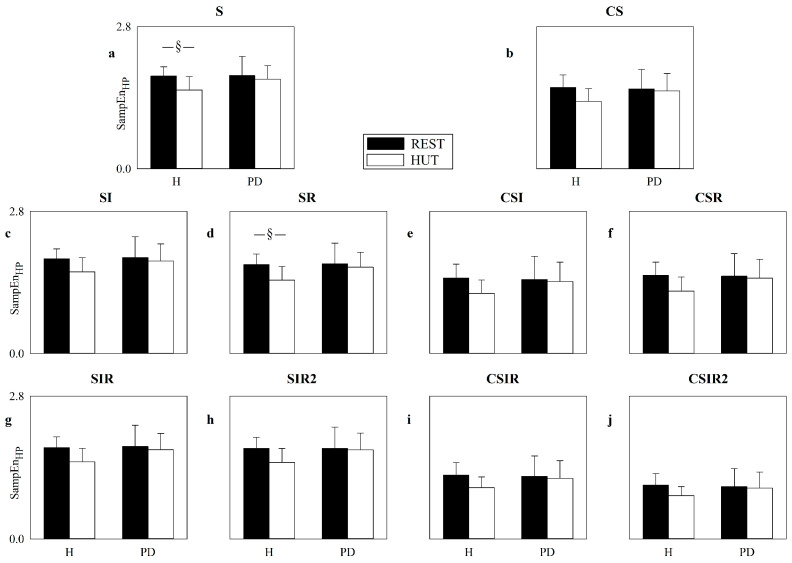
The error bar graphs show SampEn computed over HP series according to the strategies exploited to increase the number of matches, namely S (**a**), CS (**b**), SI (**c**), SR (**d**), CSI (**e**), CSR (**f**), SIR (**g**), SIR2 (**h**), CSIR (**i**) and CSIR2 (**j**). Values are given as a function of the group (i.e., H and PD) in the two considered experimental conditions, namely REST (black bars) and HUT (white bars). Data are reported as mean plus standard deviation. The symbol § indicates *p* < 0.05.

Figure 8 shows the SampEn computed over the HP series as a function of the group (i.e., H and PD) in the considered experimental conditions, namely REST (black bars) and HUT (white bars). SampEn is computed according to the strategies exploited to increase the number of matches, namely S (Figure 8a), CS (Figure 8b), SI (Figure 8c), SR (Figure 8d), CSI (Figure 8e), CSR (Figure 8f), SIR (Figure 8g), SIR2 (Figure 8h), CSIR (Figure 8i) and CSIR2 (Figure 8j). The SampEn computed according to the S strategy indicates that in the H group HUT decreased the complexity of the cardiac control, while an effect of HUT in the PD group was not found. No significant differences were detected within the same experimental condition between H individuals and PD patients, and this observation held both at REST and during HUT. The application of strategies increasing the number of matches did not lead to different conclusions and, conversely, lower statistical power was observed: indeed, only SR was able to detect the influence of HUT over the H population, while the remaining strategies did not detect any significant difference and no additional significances were detected. The VRRs of SI, SR, SIR and SIR2, averaged over all subjects regardless of the experimental condition, were 1.13, 1.08, 1.12 and 1.15, respectively, while the mean VRRs of CS, CSI, CSR, CSIR and CSIR2 were 1.15, 1.30, 1.29, 1.13 and 1.01, respectively.

**Figure 9:**
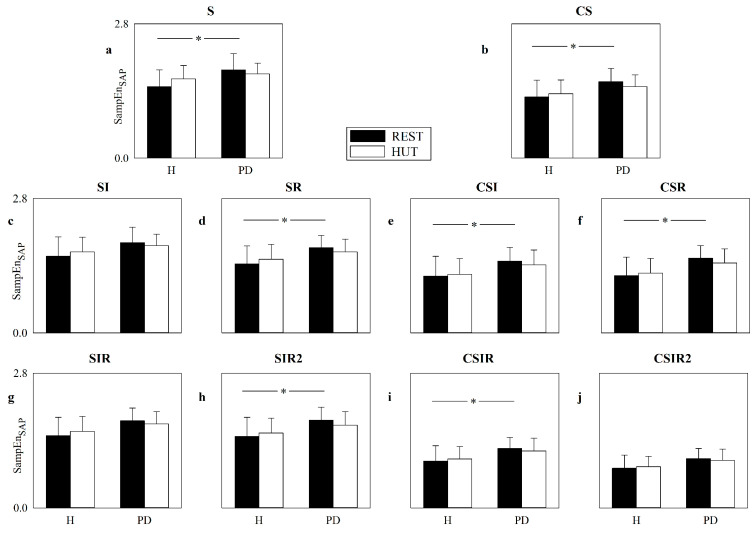
The error bar graphs show SampEn computed over the SAP series according to the strategies exploited to increase the number of matches, namely, S (**a**), CS (**b**), SI (**c**), SR (**d**), CSI (**e**), CSR (**f**), SIR (**g**), SIR2 (**h**), CSIR (**i**) and CSIR2 (**j**). Values are given as a function of the group (i.e., H and PD) in the two considered experimental conditions, namely REST (black bars) and HUT (white bars). Data are reported as mean plus standard deviation. The symbol * indicates *p* < 0.05.

Figure 9 has the same structure as Figure 8, but SampEn is computed over the SAP series. The SampEn computed according to the S strategy was able to separate the two groups at REST with values larger in PD patients than in H subjects, while this ability was lost during HUT. No significant differences between experimental conditions were found within the same group, and this observation held regardless of the group. These observations were confirmed by most of the strategies raising the number of matches, with the notable exceptions of SI, SIR and CSIR2, which were unable to detect any significant differences, thus stressing that no additional advantage in separating groups and experimental conditions was achieved. The VRRs of SI, SR, SIR and SIR2, averaged over all subjects regardless of the experimental condition, were 1.07, 1.01, 1.01 and 1.03, respectively, while the mean VRRs of CS, CSI, CSR, CSIR and CSIR2 were 0.95, 1.09, 1.03, 0.89 and 0.76, respectively.

## 6. Discussion

The main methodological findings of the study can be summarized as follows: (i) the influence of the strategies favoring pattern matching over SampEn varied with the transformation exploited, type of dynamics and level of noise superposed on the data; (ii) the application of the strategies for increasing the number of matched patterns could lead to a reduction in the range of the SampEn values; (iii) the reduction in the SampEn variance was more evident over linear stochastic series and using transformations over centered patterns; (iv) strategies increasing the number of matches could lead to an overestimation of the level of regularity despite the stochastic nature of the series or the presence of noise; (v) the ability to detect nonlinear dynamics was generally poorly affected by the application of a pattern matching strategy.

The main experimental findings of the study can be summarized as follows: (i) over both HP and SAP series, the impact of the adopted pattern matching strategies on SampEn depended on the exploited transformations; (ii) when the pattern matching strategy was applied to centered patterns, SampEn was significantly reduced compared to the standard application of SampEn, and this result held regardless of the series; (iii) strategies designed to raise the number of matches did not provide any additional advantage compared to the standard application of SampEn in separating group and experimental conditions, and this observation held for the assessment of the complexity of both cardiac and vascular controls.

### 6.1. On the Rationale of Transformations Favoring Pattern Matching

The application of transformations to the current pattern before assessing its similarity to the reference pattern has been suggested to improve pattern similarity in the computation of fuzzy entropy [29]. Among the transformations proposed to be useful to increase pattern matching, there are inversion, reversal and inversion after reversal applied to the current pattern [29], as well as centering of both current and reference patterns about their local mean [30,31]. The application of strategies for increasing pattern similarity was proposed as a practical approach to increase the robustness of the fuzzy entropy without enlarging series length [29,30,31]. It has been suggested that an augmented pattern similarity limits the random fluctuations of fuzzy entropy over short data sequences, thus decreasing the variance of fuzzy entropy estimates. Remarkably, transformations increasing pattern similarity could be applied not only to fuzzy entropy [29,30,31] but also to refined fuzzy entropy [22] and distribution entropy [42] and, more generally, to any CE metric based on the assessment of the distance between current and reference patterns [8,9,10,11,43]. These transformations could be exploited in multiscale [44,45] and multivariate [46,47] analyses as well.

### 6.2. Strategies Increasing the Number of Matches Might Lead to Misleading Conclusions over Simulated Series

The most relevant drawback of the techniques devised to increase the number of matches is that they mix different phases of the dynamic and merge classes of features that might indicate very peculiar behaviors. For example, if a pattern specifically typifies the ascending part of the positive half cycle of the sine curve, and the reverse pattern characterizes the descending part of the positive half cycle, the inverse pattern is found in the descending part of the negative half cycle and the inverted reverted pattern lies on the ascending part of the negative half cycle (see also Figure 1a–d). The consequence of considering as matches some, or all, of these patterns becomes particularly dramatic in the presence of nonlinear and deterministic dynamics, when irreversible patterns and/or phase-locked features might require distinction among the different phases of the dynamic to accurately predict specific behaviors, thus leading to the inability to fully reduce the uncertainty carried by the future values given past samples and increasing CE compared to the standard estimation of SampEn. The direct consequence is that, in the presence of deterministic, largely predictable, dynamics, higher values of complexity might be found due to the loss of the ability to predict future behaviors in relation to mixing phases. Conversely, in the presence of stochastic, largely unpredictable, dynamics, lower values might be found when the number of matches cannot notably vary while the pattern length enlarges because the number of matches is close to the maximum limit allowed by the series length. As a result, SampEn estimated by exploiting strategies artificially increasing the number of matches might span a more limited range of values compared to traditional SampEn, and this reduced interval limits the possibility of separating different types of dynamics. However, these limitations do not importantly reduce the ability to detect nonlinear dynamics via a surrogate approach, mainly because the same strategy for increasing the number of matches is applied to both original and surrogate data. We remark that the different performances of the adopted transformations require the clear indication of which technique is applied to augment the pattern matching rate.

### 6.3. Strategies Increasing the Number of Matches Are of Limited Utility in the Assessment of Cardiac and Vascular Controls

The computation of SampEn assessed according to the usual pattern matching strategy (i.e., S) confirmed that (i) complexity of the cardiac control decreased during HUT compared to REST in H subjects [11,34,43] while it remained high in PD patients [34]; (ii) complexity of the vascular control was higher at REST in PD patients compared to H subjects [34]. These conclusions were confirmed by the strategies that artificially increased the number of matches. Indeed, the detected trends were similar to SampEn computed using the standard pattern matching approach. Moreover, our data suggest that strategies designed to increase the number of matches might lead to a decrease in the statistical power of SampEn compared to the pattern matching approach exploited in traditional computation of SampEn (i.e., the S one). Indeed, we observed that some strategies were not able to detect any significant differences between groups and/or experimental conditions. This result seems to be surprising, given that, over simulations, the application of strategies increasing the number of matches tended to decrease the variance in SampEn, and this effect was especially evident when the adopted transformations operated over centered patterns and when the dynamics were dominated by stochastic components. The decrease in SampEn variance should increase the statistical power and favor differentiation among experimental conditions and/or groups of subjects. Conversely, the expected decrease in SampEn variance after the application of transformations designed to increase pattern matching rate was not observed in real HP and SAP variability data (only CSIR and CSIR2 strategies reduced SampEn variance when applied to SAP series). Thus, the general decrease in statistical power is to be attributed to the concomitant reduction in the SampEn mean (only the SampEn computed according to the SI strategy increased compared to S) in the presence of similar, or slightly increased, SampEn variances. Moreover, it cannot be excluded that the limited ability of the considered strategies might be the consequence of shuffling nonlinear features and mixing phases of the rhythmical fluctuations as suggested by simulations.

## 7. Conclusions

We do not recommend the use of strategies artificially increasing the number of matches because of the inherent risk of destroying peculiar features of the dynamic, as suggested by simulations, and the null practical advantage, as suggested by the application to the analysis of the short-term cardiac and vascular neural controls. Given the inherent stochastic nature of the physiological series, the use of strategies increasing pattern matching rate is likely to underestimate their actual complexity, and this underestimation might offset the advantage that might be linked to the reduction in the variance of the complexity markers. Moreover, given that complexity indexes based on the computation of SampEn strongly depend on the strategy exploited to find matches, we stress the importance of clearly reporting it to favor future comparisons.
